# Gut microbial immunometabolic pathways associated with cardiac arrest risk: genetic evidence and exploratory fecal microbiome profiling

**DOI:** 10.3389/fcell.2026.1931987

**Published:** 2026-10-13

**Authors:** Liuyang Xie, Yuepeng Hu, Keke Du, Kecheng Liu, Shibo Yang, Yan Zhou, Qiang Zhang, Chao Lan

**Affiliations:** 1 Department of Emergency Medicine, The First Affiliated Hospital of Zhengzhou University, Zhengzhou, China; 2 Henan Provincial Engineering Research Center for Cardiopulmonary Cerebral Resuscitation, Zhengzhou, China; 3 Henan Engineering Research Center for Cardiopulmonary and Cerebral Resuscitation, The First Affiliated Hospital of Zhengzhou University, Zhengzhou, China

**Keywords:** cardiac arrest, gut microbiota, immune cells, inflammatory factors, mediation analysis, mendelian randomization, metabolites

## Abstract

**Background:**

Cardiac arrest remains difficult to predict, and upstream biological determinants are poorly defined. Gut microbiota can influence host metabolism, immunity and inflammation, but their relevance to cardiac arrest risk remains unclear.

**Methods:**

We performed bidirectional two-sample Mendelian randomization to prioritize associations between gut microbial traits and cardiac arrest. Two-step mediation analyses were then used to assess whether circulating metabolites, immune cell phenotypes and inflammatory proteins could represent candidate intermediary pathways. An independent exploratory fecal 16S rRNA cohort of patients with cardiac arrest and healthy controls was analysed to provide descriptive post-arrest microbiome context.

**Results:**

Mendelian randomization prioritized 14 candidate gut microbial taxa or microbial metabolic pathways associated with cardiac arrest risk. Mediation analyses identified 55 candidate mediation pathways involving 41 candidate mediators, including 10 pathways with comparatively stronger statistical support based on the joint-significance criterion and the Sobel test. Metabolite-related signals accounted for most candidate mediation pathways, with additional contributions from immune cell phenotypes and inflammatory factors. In the exploratory 16S cohort, diversity and ordination patterns did not show robust group separation, whereas descriptive compositional summaries showed partial taxonomic overlap with signals highlighted by the genetic analyses; these observations were not considered directional or causal validation.

**Conclusion:**

These findings prioritize an immunometabolic framework linking gut microbial variation to cardiac arrest risk, with metabolic mediation representing the predominant candidate layer for follow-up study. The exploratory clinical 16S results provide descriptive post-arrest context rather than independent validation, and the genetic findings should be interpreted as candidate associations requiring larger prospective and experimental confirmation.

## 1 Introduction

Cardiac arrest (CA) remains one of the most lethal cardiovascular emergencies and continues to impose a major global health burden, with an annual incidence ranging from 55 to 113 cases per 100,000 population (Virani et al., 2021). Although advances in resuscitation and post-arrest care have improved acute management, prevention remains limited because many individuals at risk are not recognized before the event occurs. Current risk assessment still relies largely on structural heart disease and conventional cardiovascular risk factors, which do not fully capture the upstream biological processes that may predispose to CA. CA has multiple causes and is strongly linked to a range of cardiovascular and metabolic conditions, such as coronary artery disease, cardiomyopathy, heart failure, arrhythmias, hypertension, diabetes mellitus, dyslipidemia, obesity, and smoking (Adabag et al., 2010; Bagnall et al., 2016; Chugh et al., 2004). Therefore, identifying novel upstream risk factors and clarifying modifiable biological mechanisms are essential for improving CA risk stratification, prevention, and therapeutic intervention.

In recent years, increasing evidence has demonstrated that the gut microbiota, as a key component of the human microbial ecosystem, plays an essential role in maintaining host health and contributing to disease development. The gut microbiota continuously interacts with the host through metabolic, immune, and inflammatory pathways, thereby regulating diverse physiological and pathological processes. Previous studies have shown that gut microbial dysbiosis is associated with a broad spectrum of disorders, including metabolic diseases, neurological disorders, systemic inflammation, and cardiovascular diseases (Belizário et al., 2018; Kim, 2018; Kim et al., 2017; Ullah et al., 2023; Yang and Cong, 2021). Moreover, gut microbiota-derived bioactive metabolites, such as short-chain fatty acids, bile acids, and tryptophan-derived metabolites, have been shown to modulate immune homeostasis, inflammatory responses, and metabolic regulation (Agus et al., 2018; Al Bander et al., 2020; Boulangé et al., 2016; Mendes et al., 2019; Wu et al., 2025). Collectively, these findings suggest that gut microbiota–host interactions may contribute to disease pathogenesis through complex metabolic and immunological mechanisms.

In cardiovascular disease, these gut-host interactions have been linked to atherosclerosis, heart failure, and broader cardiometabolic dysfunction (Fan and Pedersen, 2021; Jie et al., 2017; Tang et al., 2017). These observations raise the possibility that gut microbial variation may also contribute to biological states that increase vulnerability to CA.

However, direct evidence connecting gut microbiota to CA remains limited. Existing cardiovascular studies suggest that gut-derived metabolites, intestinal barrier dysfunction, microbial translocation, and systemic immune-inflammatory activation may influence vascular biology, myocardial energetics, arrhythmia susceptibility, and responses to ischemia-reperfusion injury. Evidence involving coronary artery disease, heart failure, atrial fibrillation, and sudden cardiac death provides biological context, but cannot be treated as direct causal evidence for CA susceptibility. Most available studies are observational, clinically heterogeneous, and difficult to separate from reverse causation, because acute illness, medications, and post-arrest physiology can themselves alter the microbiome. In addition, even when gut microbial differences are observed, the intermediary host pathways that might connect microbial variation to CA risk remain poorly resolved. This challenge is especially important for CA, where prospective sampling before the event is difficult and mechanistic inference from human cohorts alone is inherently limited (Davies et al., 2018).

Mendelian randomization (MR) is a genetic epidemiological approach that uses genetic variants as instrumental variables to prioritize potential relationships between exposures and disease outcomes (Davey Smith and Hemani, 2014). In the context of CA, prospective investigations before disease onset are challenging due to its sudden occurrence and high mortality. Therefore, MR provides a useful framework for evaluating whether genetically predicted gut microbial traits are associated with CA susceptibility, while the resulting estimates still require cautious interpretation and independent biological validation.

Given the important roles of the gut microbiota in metabolic regulation, immune modulation, inflammatory responses, bile acid metabolism, and oxidative stress, we hypothesized that genetically determined alterations in gut microbial taxa and microbial metabolic pathways may be associated with CA susceptibility through the microbiota–metabolism–immunity axis. Specifically, we proposed that gut microbiota-related metabolic remodeling and immune-inflammatory dysregulation may contribute to CA-related biological processes, including cellular stress responses, metabolic imbalance, and inflammatory activation. Accordingly, we applied an integrative bidirectional two-sample MR framework to prioritize potential relationships between gut microbiota and CA, followed by two-step mediation analyses to assess candidate intermediary pathways involving circulating metabolites, immune cell phenotypes, and inflammatory proteins. Furthermore, exploratory fecal 16S rRNA microbiome profiling was conducted in an independent small clinical cohort to provide complementary descriptive context for the genetic findings and to generate hypotheses for future experimental investigation.

## 2 Materials and methods

### 2.1 Study design

This study employed an integrative bidirectional two-sample Mendelian randomization framework to prioritize potential relationships between gut microbiota and cardiac arrest and to explore candidate metabolic, immune, and inflammatory pathways. In the forward analyses, gut microbiota traits were considered as exposures and CA was treated as the outcome. Reverse analyses were performed by reversing the exposure and outcome to examine potential reverse directionality, but the asymmetry in nominally significant findings was not interpreted as formal proof of causal direction. Subsequently, a two-step mediation analysis framework was applied to investigate whether circulating metabolites, immune cell phenotypes, and inflammatory factors could represent candidate mediators linking gut microbiota to CA susceptibility. Finally, exploratory fecal 16S rRNA sequencing was conducted in an independent clinical cohort to provide descriptive post-arrest microbiome context rather than direct validation of causal mechanisms. An overview of the study design and analytical workflow is shown in Figure 1.

**FIGURE 1:**
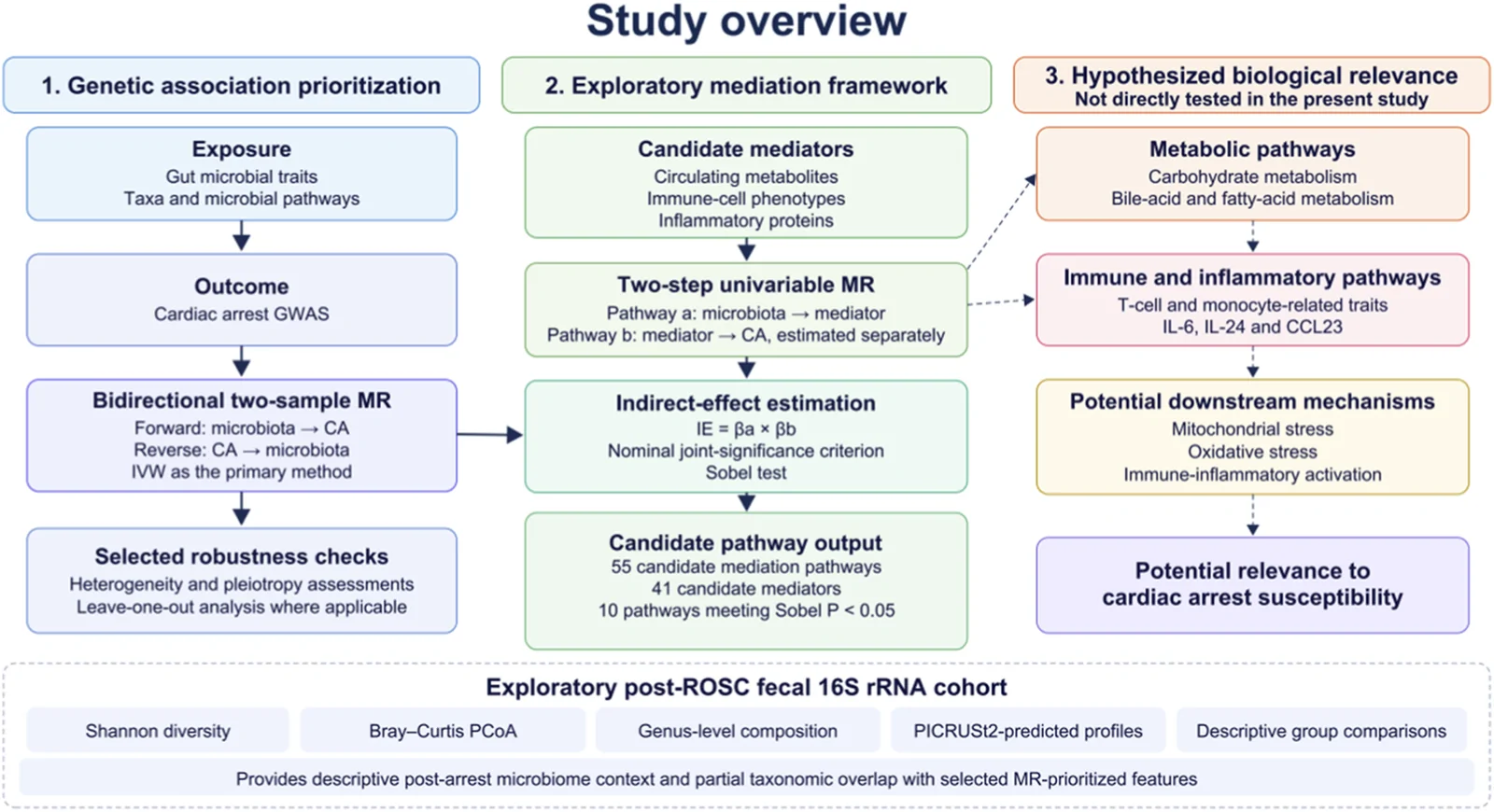
Overall study design and analytical workflow.

All MR analyses were conducted in accordance with the Strengthening the Reporting of Observational Studies in Epidemiology Using Mendelian Randomization (STROBE-MR) guidelines (Skrivankov et al., 2021).

### 2.2 Data sources

All data used for the genetic analyses in this study were obtained from publicly available genome-wide association study (GWAS) summary statistics. The study also included an independent exploratory clinical fecal 16S rRNA cohort, which is described separately below.

Gut microbiota-related GWAS data were sourced from the study by Lopera-Maya et al. (Lopera-Maya et al., 2022), with a total of 7,738 participants. This dataset contained 412 gut microbiota-related traits, including 207 taxonomic features across five phyla, 10 classes, 13 orders, 26 families, 48 genera, and 105 species, as well as 205 metabolic pathways reflecting microbial composition and functional activity.

GWAS summary statistics for cardiac arrest were obtained from the FinnGen release 11 dataset through the IEU OpenGWAS database. The phenotype was identified using endpoint code I9_CARDARR. The exact GWAS identifier, sample size, ancestry information, and phenotype definition are provided in Supplementary Table S1. Because this phenotype may include clinically heterogeneous cardiac arrest events, the present analysis was interpreted as reflecting genetic liability to cardiac arrest risk rather than a specific arrhythmic subtype or cell-specific endpoint.

GWAS summary statistics for plasma metabolites and metabolite ratios were obtained from the study by Chen et al. (Chen et al., 2023) and the NHGRI-EBI GWAS Catalog (GCST90199621–GCST90201020). The source study included 1,091 plasma metabolites and 309 metabolite ratios measured in up to 8,299 participants of European ancestry from the Canadian Longitudinal Study on Aging.

GWAS summary statistics for immune cell phenotypes were taken from the study by Orrù et al. (Orrù et al., 2020), which included 3,757 individuals of European ancestry with no overlapping samples between cohorts. This dataset included 609 immune cell traits, such as absolute cell counts, relative cell counts, cell surface antigen expression levels, and morphological parameters. These immune phenotypes covered multiple immune cell populations and differentiation stages, including B cells, conventional dendritic cells, T cells, monocytes, myeloid cells, TBNK cells, and regulatory T cells.

Inflammatory factor-related GWAS data were obtained from the study by Zhao et al. (Zhao et al., 2023), which included 91 inflammation-related plasma proteins based on protein quantitative trait locus (pQTL) analyses. Detailed information on all data sources is provided in Supplementary Table S1.

### 2.3 Selection of instrumental variables

Genetic variants were selected as instrumental variables (IVs) according to the three core assumptions of MR analysis (Supplementary Figure S1).

For metabolites, immune cells, and inflammatory factors, single nucleotide polymorphisms (SNPs) significantly associated with the exposures were selected using the genome-wide significance threshold of *P* < 5 × 10^−8^. Given that only a limited number of SNPs associated with gut microbiota traits achieved genome-wide significance, a more relaxed threshold of *P* < 1 × 10^−5^ was adopted for gut microbiota-related SNP selection.

To ensure the independence of selected SNPs, linkage disequilibrium (LD) clumping was performed with *r* ^2^ < 0.001 and a clumping distance of 10,000 kb. Subsequently, harmonization was conducted between exposure and outcome datasets to ensure consistency in effect alleles and effect directions, and palindromic SNPs were excluded.

We calculated the proportion of phenotypic variance explained by each SNP (R^2^) and the F statistic to assess instrument strength and reduce the risk of weak-instrument bias (Burgess et al., 2017). SNPs with F-statistics <10 were excluded. R^2^ was calculated as follows:

$$R^{2}=2\times \left(1-\text{MAF}\right)\times \text{MAF}\times \beta^{2}$$

where MAF represents the minor allele frequency and β represents the effect size of the exposure (Mackinnon et al., 2002). The exposure effect estimate β was used on the scale reported in the corresponding exposure GWAS. Instrument strength was assessed at the SNP level using the available minor allele frequency, exposure effect estimate, and exposure-GWAS sample size. The corresponding F statistic was calculated as F = R^2^(N − 2)/(1 − R^2^), where N denotes the sample size of the exposure GWAS.

### 2.4 Mendelian randomization analysis

Bidirectional two-sample MR analyses were performed in R (version 4.3.1) using the TwoSampleMR package to investigate genetically predicted associations among gut microbiota, metabolites, immune cell phenotypes, inflammatory factors, and cardiac arrest.

In univariable Mendelian randomization (UVMR) analyses, selected instrumental variables were first aligned with the outcome GWAS summary data to match effect directions. The inverse variance weighted (IVW) method using a random-effects model served as the primary approach to estimate associations. Weighted median and weighted mode methods were also applied as complementary sensitivity approaches.

Heterogeneity and horizontal pleiotropy were assessed using Cochran Q test and MR-Egger regression, respectively. Associations were prioritized when the IVW analysis reached the prespecified nominal threshold and the effect direction was broadly consistent across complementary MR methods. Given the high-dimensional screening framework, nominal significance was not interpreted as definitive causal evidence.

The mediation component used a two-step univariable MR framework. Pathways a and b were estimated separately using genetic instruments for the microbial exposure and mediator, respectively. The microbial exposure and mediator were not simultaneously included in a multivariable MR model.

### 2.5 Mediation analysis

A two-step MR mediation framework was used to prioritize candidate indirect pathways linking gut microbial traits with CA through circulating metabolites, immune cell phenotypes, or inflammatory factors. The resulting indirect effects were interpreted as exploratory candidate mediation signals rather than definitive mechanistic mediation.

Mediation analysis was based on three statistical components. Pathway a represented the genetically predicted association between each microbial exposure and candidate mediator (βa). Pathway b represented the genetically predicted association between the mediator and cardiac arrest (βb), estimated using univariable two-sample MR with genetic instruments for the mediator. The microbial exposure was not included simultaneously in pathway b; therefore, βb was not interpreted as an exposure-adjusted direct effect. Pathway c represented the total exposure–outcome association (β_total). Genetic instruments for the microbial exposure and mediator were analyzed separately and were not pooled into a common multivariable instrument set. The indirect effect (IE) was calculated using the product-of-coefficients method as IE = βa × βb. The covariance between βa and βb was assumed to be zero because the two coefficients were estimated separately. The standard error of the indirect effect was estimated using the second-order Delta method (Aroian formula): SE_IE = √(βa^2^SEb^2^ + βb^2^SEa^2^ + SEa^2^SEb^2^). Approximate 95% confidence intervals were calculated as IE ± 1.96 × SE_IE, and two-sided P values were obtained using the corresponding normal approximation. The mediation ratio was calculated as IE/β_total × 100%. This ratio was interpreted as a conventional proportion mediated only when the indirect and total effects were directionally concordant. When their directions differed, a negative ratio was described as an inconsistent mediation or suppression pattern and was not interpreted as the percentage of the total effect explained by the mediator. The joint-significance criterion required nominal statistical significance for both pathway a and pathway b (P < 0.05) and was used for exploratory screening. Because the 55 retained pathways were selected from a larger upstream screening space, correction restricted to these 55 pathways would not fully account for all initial exposure–mediator tests. Accordingly, all pathway-level findings were interpreted as exploratory and hypothesis-generating.

### 2.6 Sensitivity analysis

Sensitivity analyses were performed for the underlying MR associations where applicable. Comprehensive MR-Egger, weighted-median, and leave-one-out analyses were not performed for every exposure–mediator–outcome combination because of the large number of candidate pathways. Therefore, the mediation results should be interpreted as candidate signals rather than fully sensitivity-validated pathways.

A key assumption of MR analysis is that instrumental variables influence the outcome only through the exposure of interest. Therefore, horizontal pleiotropy was checked using the intercept obtained from MR-Egger regression. An intercept estimate close to zero with a non-significant P value was interpreted as no detected evidence of directional horizontal pleiotropy, although the limited statistical power of this test was acknowledged.

Leave-one-out analyses were used for selected underlying MR associations to assess sensitivity to individual instruments. These analyses were not interpreted as proof that pleiotropy or other biases were absent from the complete mediation network.

### 2.7 Exploratory fecal 16S rRNA sequencing cohort

To provide exploratory biological context for the MR framework, fecal samples were collected from an independent exploratory clinical cohort comprising patients with cardiac arrest (CA, n = 5) and healthy controls (Non-CA, n = 6) at the First Affiliated Hospital of Zhengzhou University for 16S rRNA gene sequencing analysis. This exploratory cohort was not designed for formal hypothesis testing, and no formal sample size calculation was performed. Because CA samples were collected within 48 h after return of spontaneous circulation (ROSC), the 16S data were interpreted as descriptive post-arrest microbiome context rather than evidence of pre-arrest risk or causal direction. Future validation will require substantially larger, prospectively assembled cohorts.

Fecal samples were removed from frozen storage and processed immediately. An appropriate amount of each sample was transferred into a centrifuge tube containing lysis buffer and homogenized using a Tissuelyser-48 multi-sample tissue grinder (Shanghai Jingxin Industrial Development Co., Ltd., Shanghai, China) at 60 Hz. Microbial DNA was subsequently extracted using the MagBeads FastDNA Kit for Soil (catalog no. 116564384; MP Biomedicals, CA, United States) according to the manufacturer’s instructions. DNA integrity was assessed by 0.8% agarose gel electrophoresis, and DNA concentration and purity were measured using a NanoDrop spectrophotometer. The V3–V4 region of the bacterial 16S rRNA gene was amplified using the primers CCTAYGGGRBGCASCAG and GGACTACNNGGGTATCTAAT. Amplicon libraries were sequenced on an Illumina NovaSeq 6000 platform using a paired-end 250-bp strategy.

Raw reads were processed using QIIME 2 (release 2020.2). Paired-end reads were quality-filtered, merged, denoised, and screened for chimeric sequences using DADA2 implemented in QIIME 2, and amplicon sequence variants were inferred. Taxonomic classification was performed using the q2-feature-classifier plugin against the SILVA 138 reference database. For alpha- and beta-diversity analyses, the feature table was rarefied to the minimum sequencing depth across the included samples. Alpha diversity was summarized using the Shannon index, and beta diversity was evaluated using Bray–Curtis distances and principal-coordinate analysis. Predicted functional profiles were generated using PICRUSt2 (version 2.5.2). Predicted KEGG Orthology features were summarized at the group level using descriptive comparisons of mean relative abundance. Given the small sample size and exploratory nature of the analysis, these functional comparisons were not interpreted as confirmatory hypothesis-testing results.

The human-participant component of this study was reviewed and approved by the Ethics Committee of the First Affiliated Hospital of Zhengzhou University (approval no. 2025-KY-1013-002; approved on November 21, 2025; valid until November 20, 2026). Written informed consent was obtained from all participants or their legal representatives before enrollment.

## 3 Results

### 3.1 Selection of instrumental variables

After linkage disequilibrium (LD) clumping and data harmonization, 3,842 SNPs associated with gut microbiota traits were selected as instrumental variables using the threshold of P < 1 × 10^−5^. We also identified 2,538, 2,567, and 282 SNPs significantly associated with metabolites, immune cell traits, and inflammatory factors, respectively, at the genome-wide threshold P < 5 × 10^−8^ (Supplementary Tables S2–S5).

Detailed SNP-level information for the exposure instruments is provided in Supplementary Tables S2–S5. MR estimates for the corresponding exposure categories are provided in Supplementary Tables S6–S9.

All included instruments had F-statistics >10, confirming adequate strength and reducing concern about weak-instrument bias in the subsequent MR analyses.

### 3.2 Bidirectional two-sample mendelian randomization analysis of gut microbiota and cardiac arrest

In the bidirectional Mendelian randomization analysis of gut microbiota and cardiac arrest (Figure 2), 14 gut microbial taxa and metabolic pathways were prioritized according to the inverse variance weighted method at a nominal threshold (P < 0.05). Full Mendelian randomization estimates for these candidate associations are listed in Supplementary Table S17.

**FIGURE 2:**
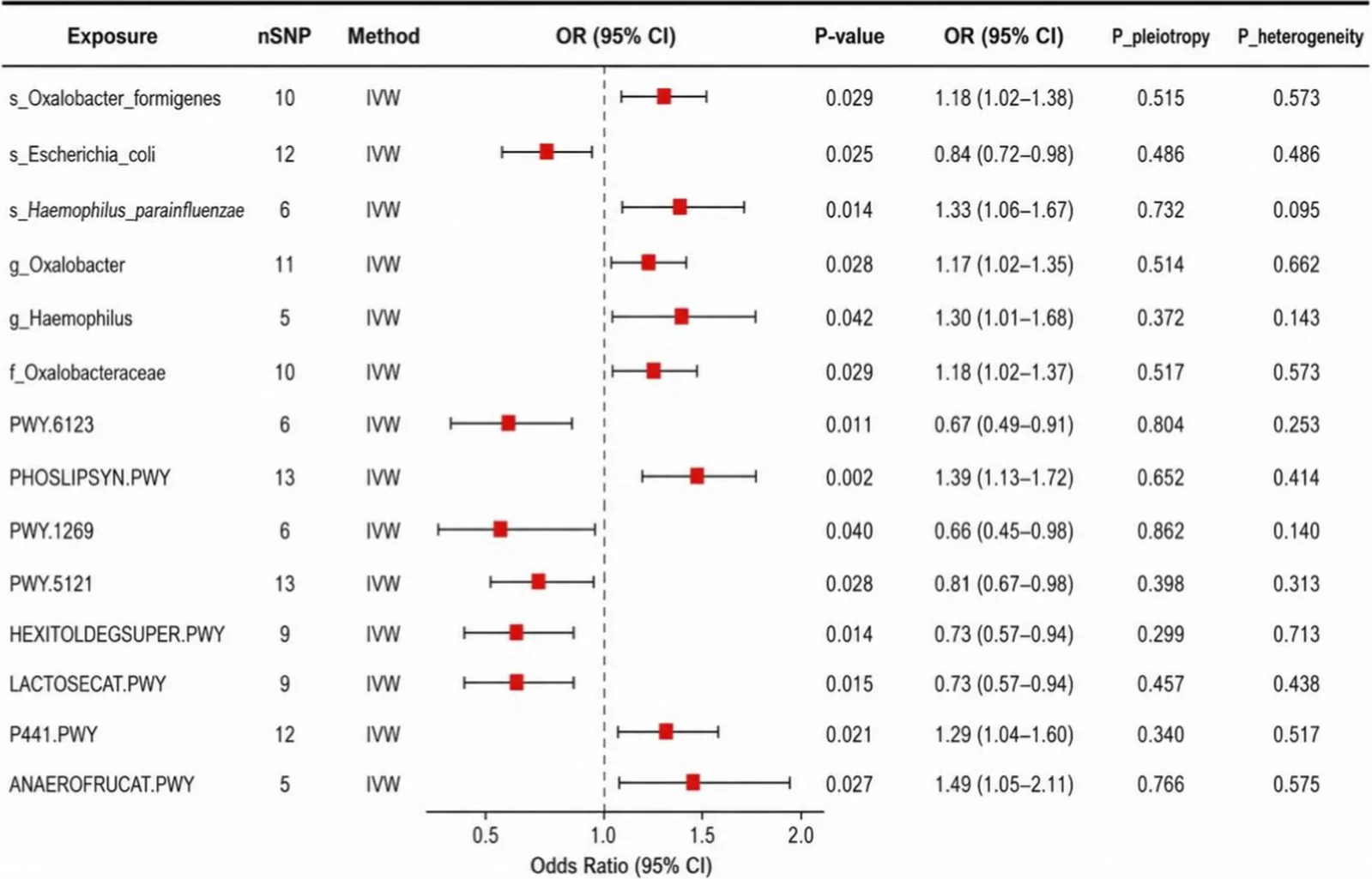
Mendelian randomization analysis of gut microbiota and cardiac arrest. Forest plot showing the odds ratios (ORs) and 95% confidence intervals (CIs) for 14 candidate microbial taxa and metabolic pathways prioritized in association with cardiac arrest. The dashed vertical line represents the null effect (OR = 1).

These microbial features showed associations in both risk-increasing and risk-decreasing directions. For example, higher genetically predicted abundance of Oxalobacter formigenes was associated with increased cardiac arrest risk (OR = 1.18, 95% CI: 1.02–1.38, P = 0.029), whereas several taxa and pathways, including *Escherichia coli*, showed OR values below 1. This pattern suggests heterogeneous candidate microbiota–host associations rather than a uniform directional effect.

Associations showing evidence of substantial horizontal pleiotropy or heterogeneity were examined cautiously. No statistically significant evidence of these features was detected for most retained associations in the applied tests. Each exposure was instrumented by 5–13 SNPs; therefore, the limited number of instruments and the power of the sensitivity tests should be considered when interpreting stability.

Reverse MR analysis was performed to evaluate genetically predicted associations of cardiac arrest liability with the gut microbial traits. Only one microbial feature reached nominal significance in the reverse analysis, compared with 14 traits prioritized in the forward analysis. This asymmetry was descriptive and did not formally establish the direction of association. Formal Steiger directionality testing was not performed; therefore, these findings cannot confirm a microbiota-to-cardiac-arrest direction or establish the assumed mediation sequence. Detailed graphical results of the reverse MR analysis are presented in Supplementary Figure S2.

### 3.3 Genetically predicted associations of circulating metabolites with cardiac arrest

We next examined genetically predicted associations between circulating metabolites and cardiac arrest. We next examined genetically predicted associations between circulating metabolites and cardiac arrest (Figure 3).

**FIGURE 3:**
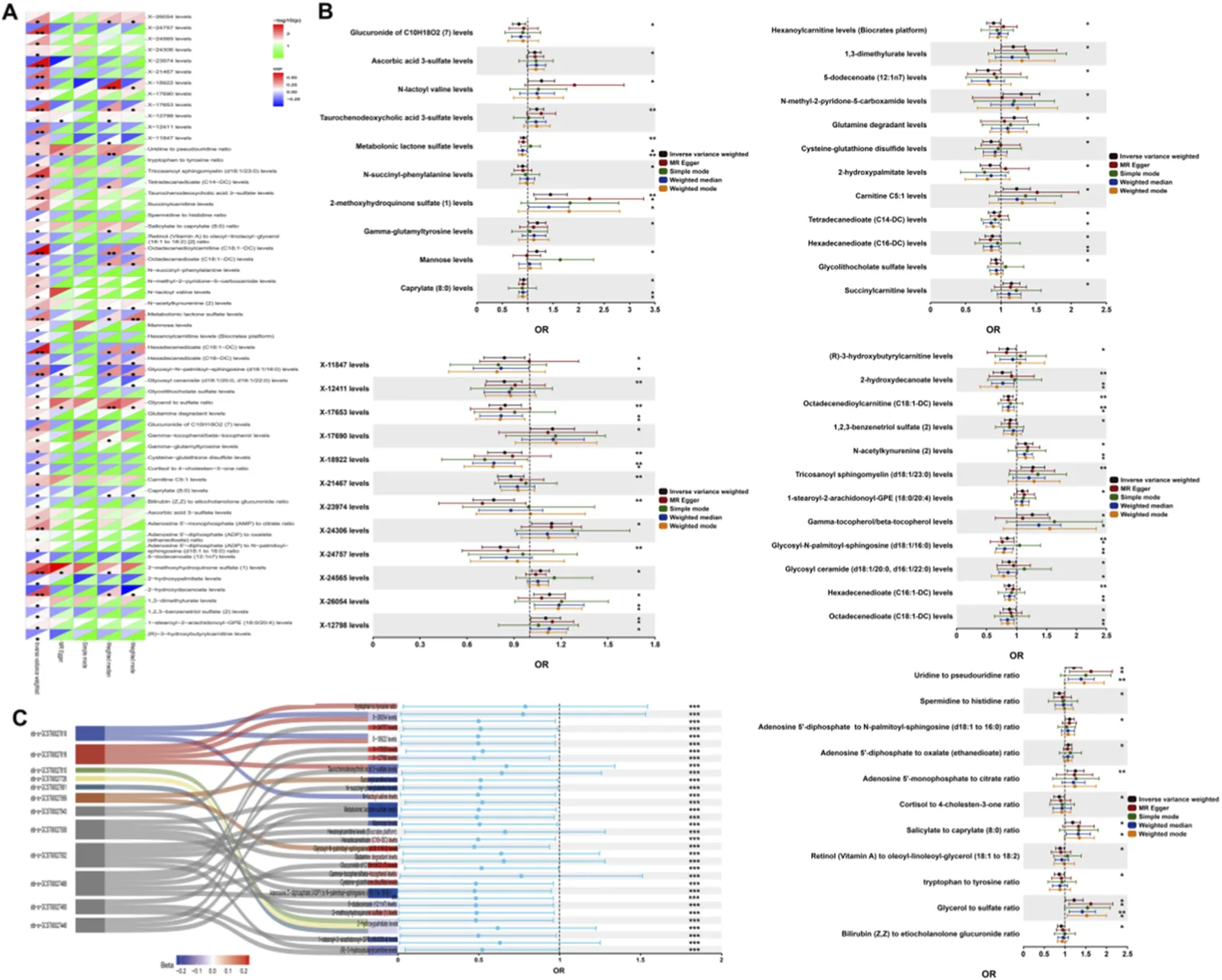
Candidate mediation analysis of metabolites in the association between gut microbiota and cardiac arrest. **(A)** Heatmap showing the associations and statistical significance of candidate metabolites across the applied Mendelian randomization methods. **(B)** Forest plots showing Mendelian randomization effect estimates, including odds ratios (ORs) and 95% confidence intervals (CIs), obtained using different analytical methods. **(C)** Sankey diagram illustrating the relationships between genetic instruments and candidate metabolites, together with the corresponding effect estimates. These findings represent candidate intermediary signals.

In the primary inverse variance weighted analysis, multiple circulating metabolites showed nominally prioritized associations with cardiac arrest risk. Several metabolites appeared to act as potential risk indicators (OR > 1), including 1,3-dimethylurate, 5-dodecenoate (12:1n7), N-methyl-2-pyridone-5-carboxamide, glutamine degradant, and cysteine-glutathione disulfide. The effect directions were consistent across the inverse variance weighted and weighted median analyses, providing supportive consistency across the applied MR methods.

Conversely, several metabolites showed inverse associations with cardiac arrest risk (OR < 1), including 2-hydroxydecanoate, octadecenedioylcarnitine (C18:1-DC), gamma-tocopherol/beta-tocopherol, and glycosyl sphingolipids. These findings indicate that specific metabolic programs may be associated with cardiac arrest risk in inverse directions.

Notably, several metabolites related to fatty acid metabolism (C14-DC, C16-DC, and C18:1-DC) and carnitine-related compounds, including succinylcarnitine and hexanoylcarnitine, showed associations with cardiac arrest risk. This pattern is compatible with altered mitochondrial fatty-acid utilization and broader energetic disturbance, but does not by itself establish a myocardial mechanism.

Sensitivity analyses using MR-Egger, weighted median, simple mode, and weighted mode methods showed broadly similar effect directions for selected signals. These analyses provide supportive robustness checks, although their power is limited when the number of instruments is small.

Several relatively understudied metabolites, such as X-11847, X-12411, and X-17653, also showed nominally prioritized associations, suggesting that additional metabolic mechanisms relevant to cardiac arrest remain to be defined.

Overall, our findings support genetically predicted links between circulating metabolites and cardiac arrest risk. In particular, disturbances in fatty acid metabolism, amino acid metabolism, and oxidative stress-related pathways appear to be closely associated with cardiac arrest, highlighting metabolic dysregulation as a candidate biological layer associated with cardiac arrest susceptibility.

### 3.4 Genetically predicted associations of immune cell phenotypes with cardiac arrest

We next evaluated genetically predicted associations between immune cell phenotypes and cardiac arrest. Multiple immune cell traits were implicated, spanning both adaptive and innate immune responses (Figure 4).

**FIGURE 4:**
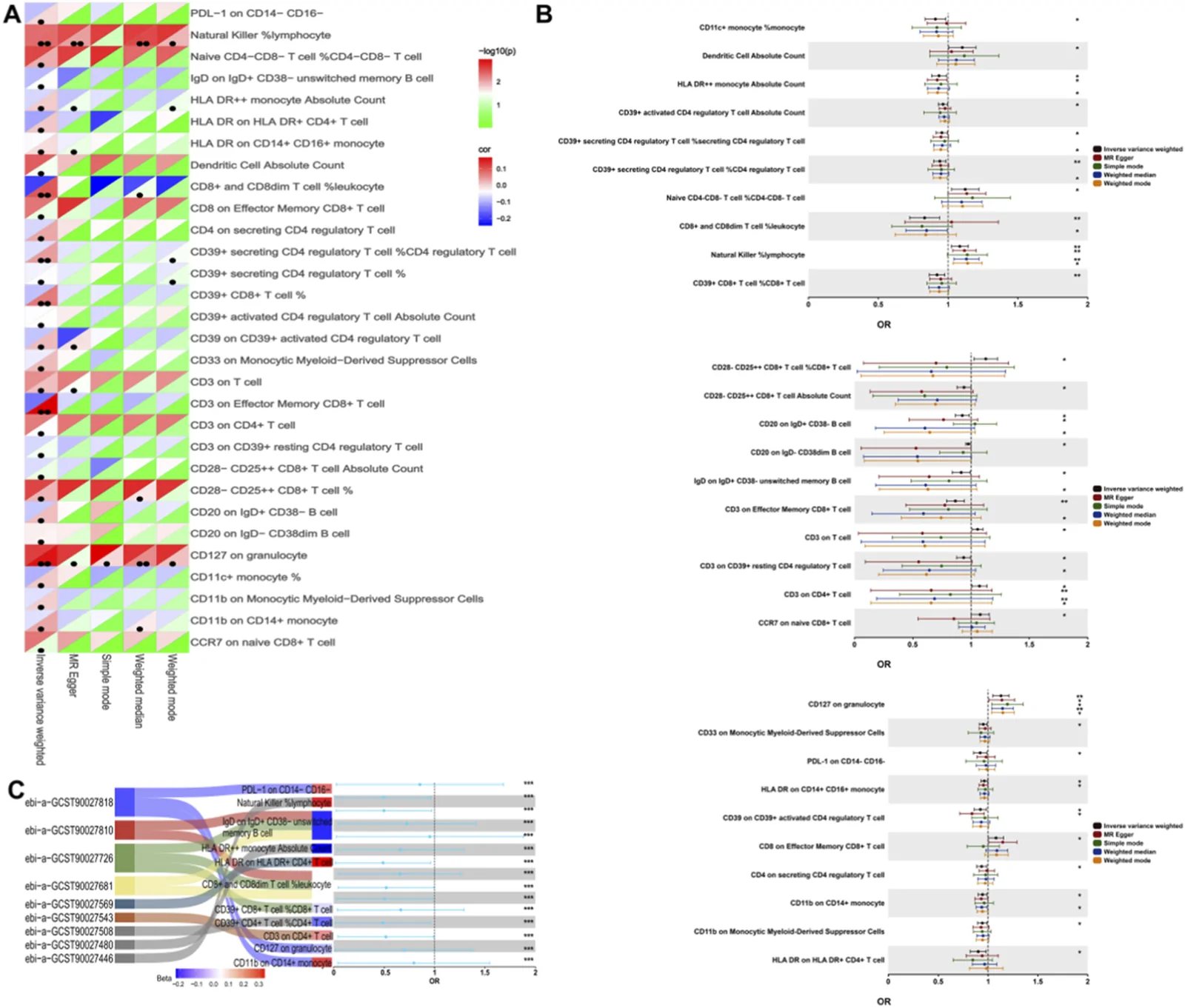
Mediation analysis of immune cell phenotypes in the association between gut microbiota and cardiac arrest. **(A)** Heatmap showing the associations and statistical significance of selected immune cell phenotypes across the applied Mendelian randomization methods. **(B)** Forest plots presenting Mendelian randomization effect estimates, including ORs and 95% CIs, for selected immune cell phenotypes. **(C)** Sankey diagram illustrating the relationships between genetic instruments and selected immune cell phenotypes, together with the corresponding effect estimates. These immune cell phenotypes were prioritized as candidate intermediary traits within the exploratory mediation framework.

Among adaptive immune phenotypes, several T-cell subsets showed genetically predicted associations with CA risk. In particular, the proportions of CD8+ T cells, CD8dim T cells (% leukocyte), and CD39+ CD8+ T cells showed consistent effect directions across MR methods. These results prioritize cytotoxic T-cell-related phenotypes for further investigation but do not demonstrate that gut microbiota directly shapes these immune states.

Among innate immune phenotypes, multiple monocyte- and dendritic-cell-related traits showed associations with CA risk. For example, the absolute count of HLA-DR++ monocytes, HLA-DR expression on CD14+ CD16+ monocytes, and total dendritic cell count yielded broadly similar results across analytical methods. These findings prioritize antigen-presentation and inflammatory-activation pathways for future study.

Several immune cell surface markers were also prioritized, including CD11b on CD14+ monocytes, CD127 on granulocytes, and PD-L1 on CD14− CD16− monocytes. These markers reflect distinct states of immune activation and immune regulation. The present analyses do not establish a complete gut microbiota → immune-cell → CA causal chain.

Sensitivity analyses indicated limited evidence of directional horizontal pleiotropy for most immune traits in the applied tests. One exception was the association between CD39 on CD39+ secreting CD4 regulatory T cells and CA, which showed evidence of horizontal pleiotropy in the MR-Egger intercept test (intercept = −0.0253, P = 0.0384). This finding should therefore be interpreted cautiously.

Immune-related signals appeared in both risk-increasing and risk-decreasing directions. This pattern indicates heterogeneity among candidate immune associations and should not be interpreted as proof of bidirectional biological regulation or as evidence that microbiota directly alter immune-cell phenotypes.

### 3.5 Genetically predicted associations of inflammation-related factors with cardiac arrest

We next performed univariable Mendelian randomization analyses to examine whether inflammatory factors were associated with cardiac arrest risk. Several inflammatory markers showed nominally prioritized associations with cardiac arrest risk (Figure 5), supporting a role for inflammatory activity within the broader network.

**FIGURE 5:**
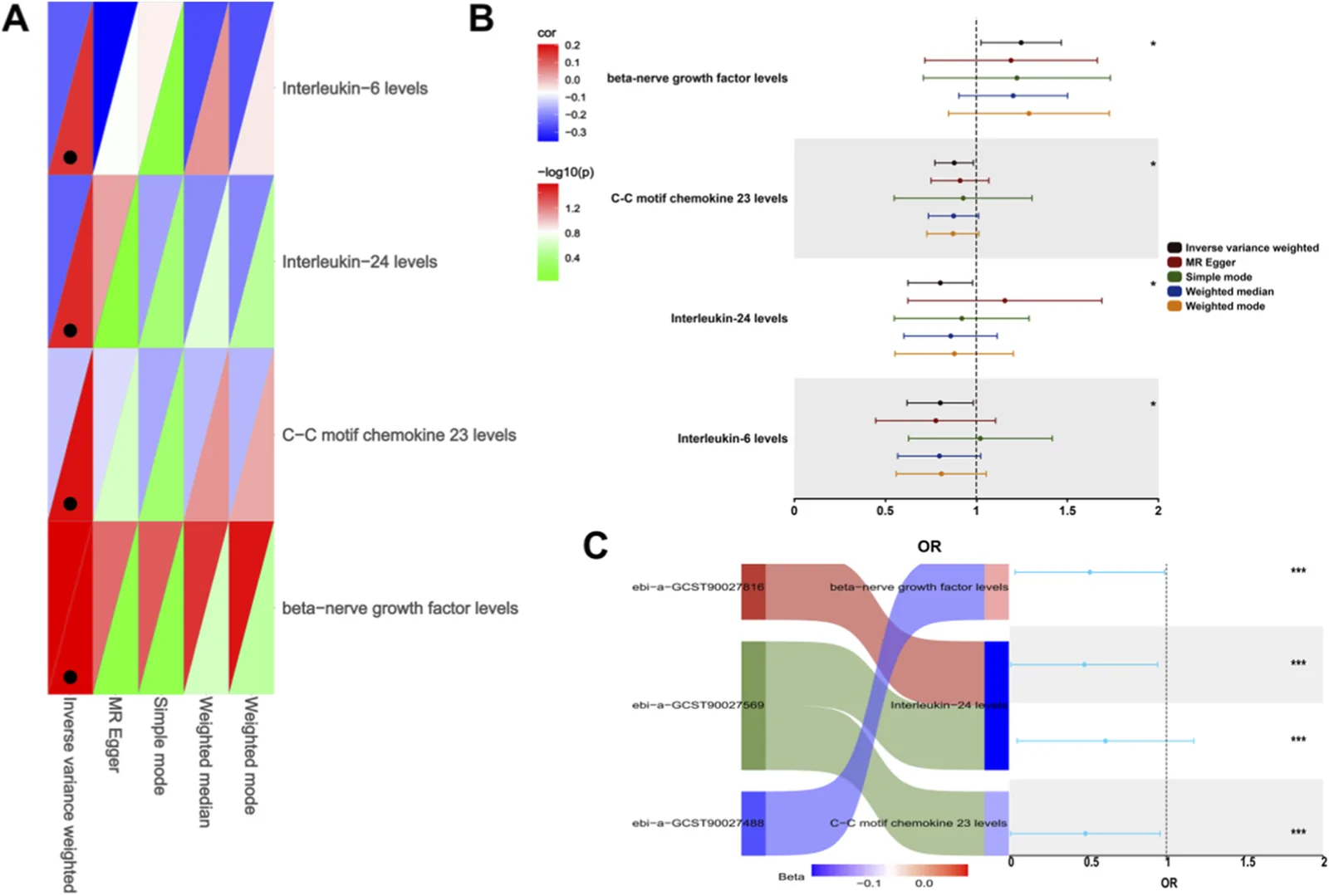
Mediation analysis of inflammation-related factors in the association between gut microbiota and cardiac arrest. **(A)** Heatmap showing the associations and statistical significance of candidate inflammatory mediators across the applied Mendelian randomization methods. **(B)** Forest plots presenting Mendelian randomization effect estimates, including ORs and 95% CIs, for candidate inflammatory mediators. **(C)** Sankey diagram illustrating the relationships between genetic instruments and candidate inflammatory mediators, together with the corresponding effect estimates. Key inflammatory factors, including interleukin-24 (IL-24), interleukin-6 (IL-6), C-C motif chemokine ligand 23 (CCL23), and beta-nerve growth factor (β-NGF), were prioritized as candidate intermediary signals within the exploratory framework.

Specifically, C-C motif chemokine 23 (CCL23), interleukin-24 (IL-24), interleukin-6 (IL-6), and beta-nerve growth factor (β-NGF) demonstrated genetically predicted associations with CA. These signals should be interpreted as candidate inflammatory pathways requiring independent validation.

We also observed that these inflammatory mediators did not act uniformly. Some appeared to increase CA risk, whereas others were associated with lower risk, highlighting the heterogeneous directions of the candidate inflammatory associations.

Sankey diagram analyses showed that multiple gut microbial taxa and microbial metabolic pathways, including *Escherichia coli*, PWY-6123, and the LACTOSECAT pathway, were statistically linked to CA through these inflammation-related factors. These results prioritize possible inflammatory intermediary pathways but do not establish a complete causal chain.

Overall, these findings prioritize inflammatory factors as candidate intermediary signals that may link gut microbial variation with CA susceptibility through immune-inflammatory pathways.

### 3.6 Sensitivity analyses

The following sensitivity analyses primarily concerned the underlying MR associations and were not available for every complete exposure–mediator–outcome pathway. To test the stability of our MR results, we performed heterogeneity and horizontal pleiotropy analyses for gut microbiota, metabolites, immune cell phenotypes, and inflammatory factors, respectively (Supplementary Tables S10–S13).

Overall, Cochran Q tests indicated no statistically significant heterogeneity in most analyses across the four categories of exposures. A small number of metabolites and immune cell traits showed evidence of heterogeneity, as detailed in Supplementary Tables S10–S13.

MR-Egger intercept tests were used to assess directional horizontal pleiotropy. Most intercept values were close to zero and not statistically significant in the applied tests. Given the limited power of these tests, non-significant results should be interpreted as no detected evidence rather than proof that pleiotropy was absent.

Only a small number of immune cell traits showed mild evidence of horizontal pleiotropy, with slightly stronger signals observed for CD39-related regulatory T-cell markers. However, these findings were not consistent across tests and should therefore be interpreted cautiously and do not permit firm conclusions regarding the absence of pleiotropic bias.

Sensitivity analyses were available for selected underlying MR associations, but comprehensive MR-Egger, weighted-median, and leave-one-out analyses were not performed for every exposure–mediator–outcome combination. Therefore, these analyses provide supportive checks for selected components but do not fully validate each individual mediation pathway.

Taken together, the applied sensitivity analyses did not identify substantial heterogeneity or directional pleiotropy for most retained underlying associations. These findings support cautious prioritization of the candidate associations but do not eliminate residual bias in the complete mediation network.

Detailed reverse Mendelian randomization results are provided in Supplementary Table S14.

### 3.7 Mediation analyses

Among the 14 microbial traits prioritized in the primary MR analysis, 12 formed at least one candidate mediation pathway meeting the nominal joint-significance criterion. PWY-1269 yielded no candidate mediator with a nominally significant exposure–mediator association, whereas the five circulating proteins initially associated with genus *Haemophilus* did not show nominally significant mediator–outcome associations. Therefore, these two microbial traits did not contribute to the final set of candidate mediation pathways. The remaining 12 microbial exposures yielded 55 candidate exposure–mediator–outcome pathways involving 41 unique mediator traits, including 27 metabolites, 11 immune-cell phenotypes, and 3 inflammatory factors.

Among these, 10 pathways met both the joint-significance criterion and the nominal Sobel criterion and therefore showed comparatively stronger statistical support within the applied exploratory mediation framework (Figure 6). This classification does not imply definitive mechanistic mediation or independence from multiple-testing concerns.

**FIGURE 6:**
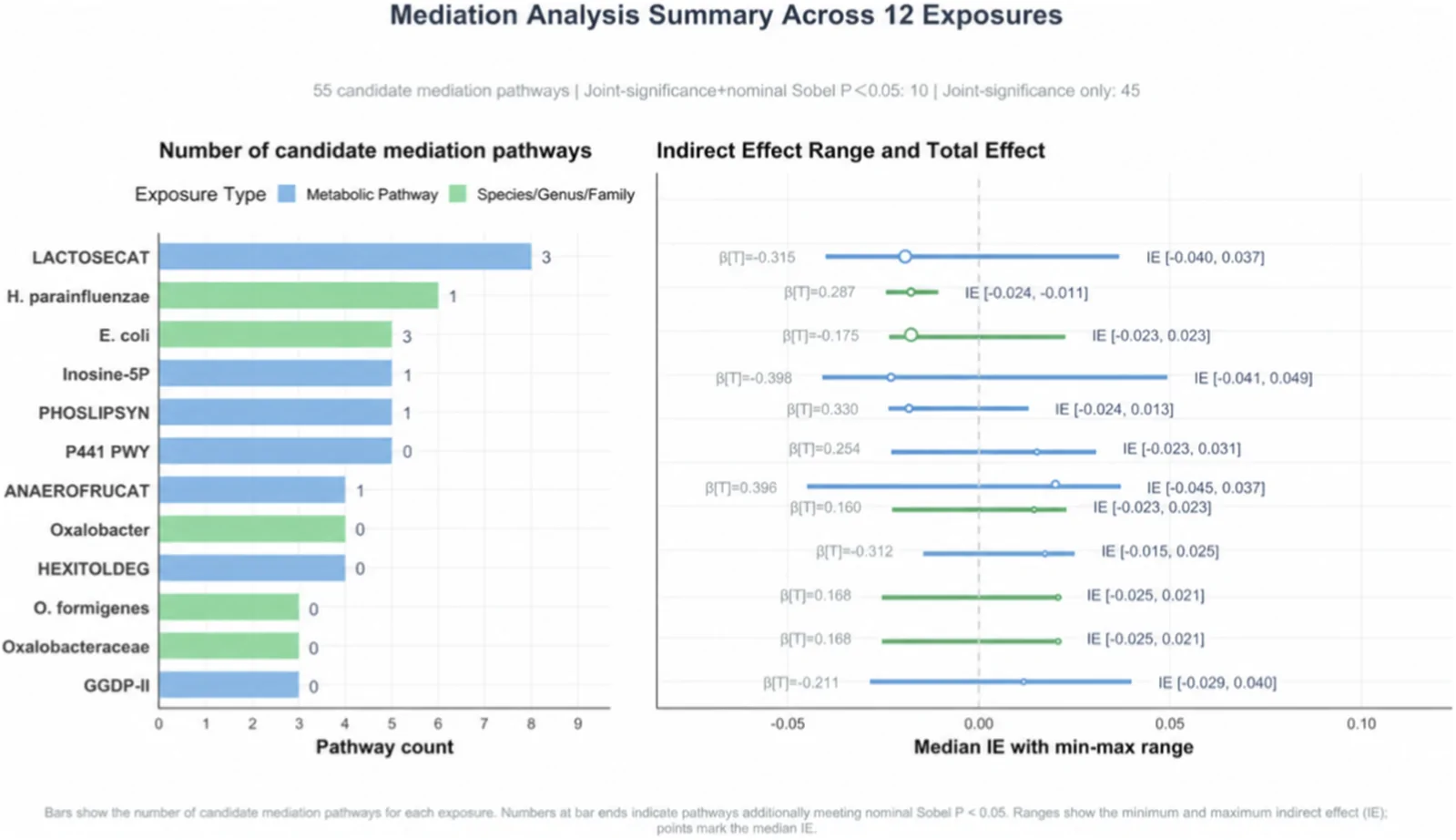
Overall structure of the exploratory mediation analysis and distribution of candidate pathways across exposures. The bar graph counts candidate mediation pathways for each exposure item. Dots and error bars represent the median indirect effects (IEs) and their corresponding ranges. In total, 55 candidate mediation pathways were screened and prioritized.

We classified all mediators into three categories. Metabolites accounted for the majority of nominally supported candidate mediation pathways and spanned the widest range of biological processes, suggesting that metabolic regulation represents the predominant candidate intermediary layer through which gut microbial variation may be related to CA risk. These results should not be interpreted as proof that the highlighted metabolites function as central biological hubs.

Immune-cell mediation analyses further showed that multiple T-cell subsets and monocyte-related traits participated in candidate mediation pathways, indicating that adaptive and innate immune phenotypes may be relevant intermediary layers. Although fewer inflammatory factors were identified than metabolites or immune markers, several inflammatory mediators appeared in multiple pathways. These findings prioritize possible regulatory bridges but do not prove a complete microbiota–immune–CA causal pathway.

At the individual pathway level, some exposures, particularly the LACTOSECAT metabolic pathway, were associated with CA through multiple candidate mediators. For example, the LACTOSECAT pathway showed a negative indirect effect through the unannotated metabolite X-17653 (IE = −0.0326, nominal Sobel P = 0.0214), whereas metabolonic lactone sulfate yielded an indirect effect in the opposite direction (IE = 0.0161, nominal Sobel P = 0.0298) (Figures 7, 8). The remaining eight pathways that additionally met the nominal Sobel P < 0.05 criterion are presented in Supplementary Figure S5. These nominal findings suggest heterogeneous candidate routes that require further validation.

**FIGURE 7:**
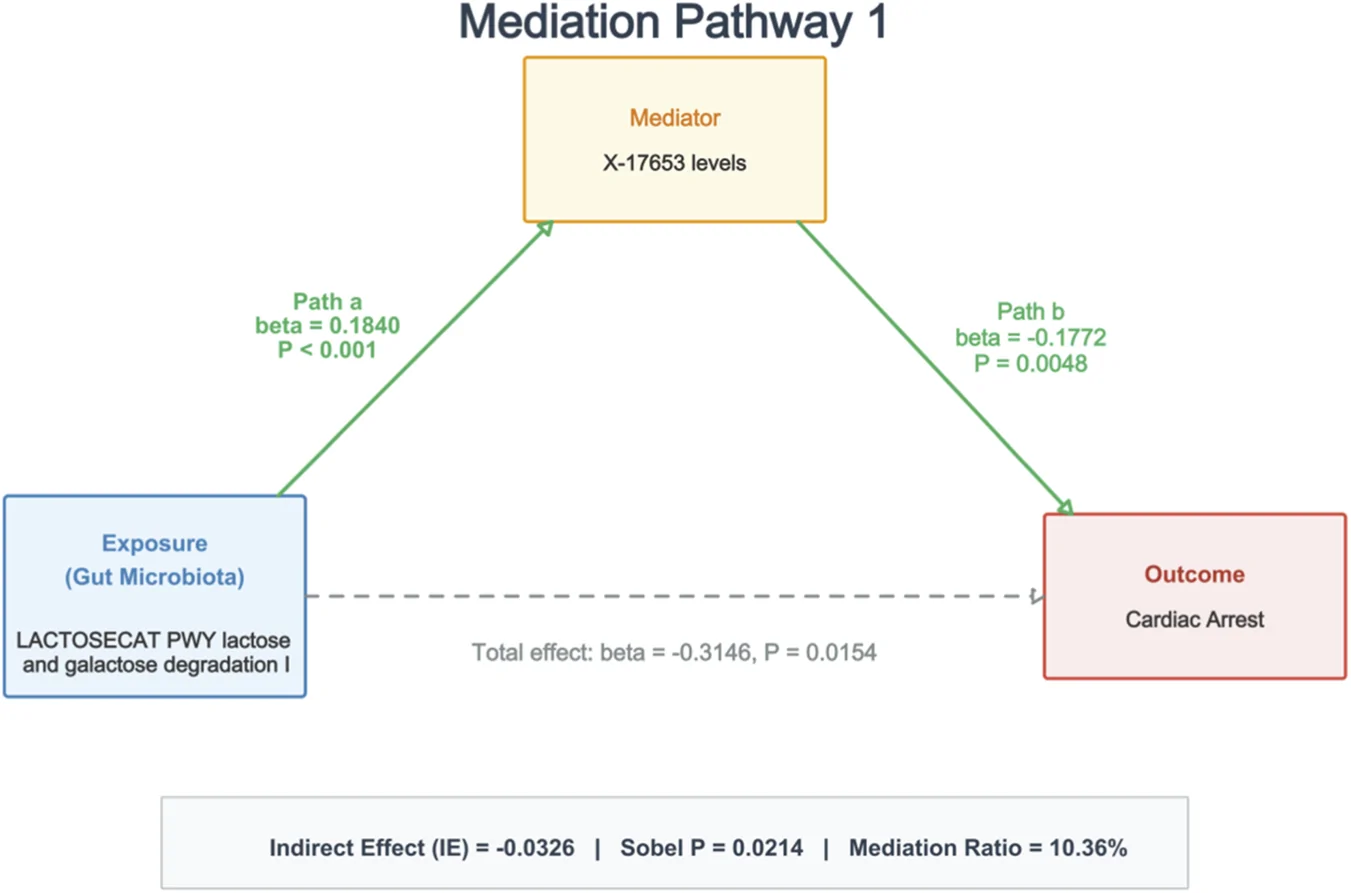
Representative exploratory mediation pathway: the LACTOSECAT microbial metabolic pathway and cardiac arrest through the unannotated metabolite X-17653. The figure presents the exposure–mediator association, mediator–outcome association, total effect, and indirect effect. This pathway showed a nominally supported negative indirect effect (IE = −0.0326, Sobel P = 0.0214) within the exploratory mediation framework.

**FIGURE 8:**
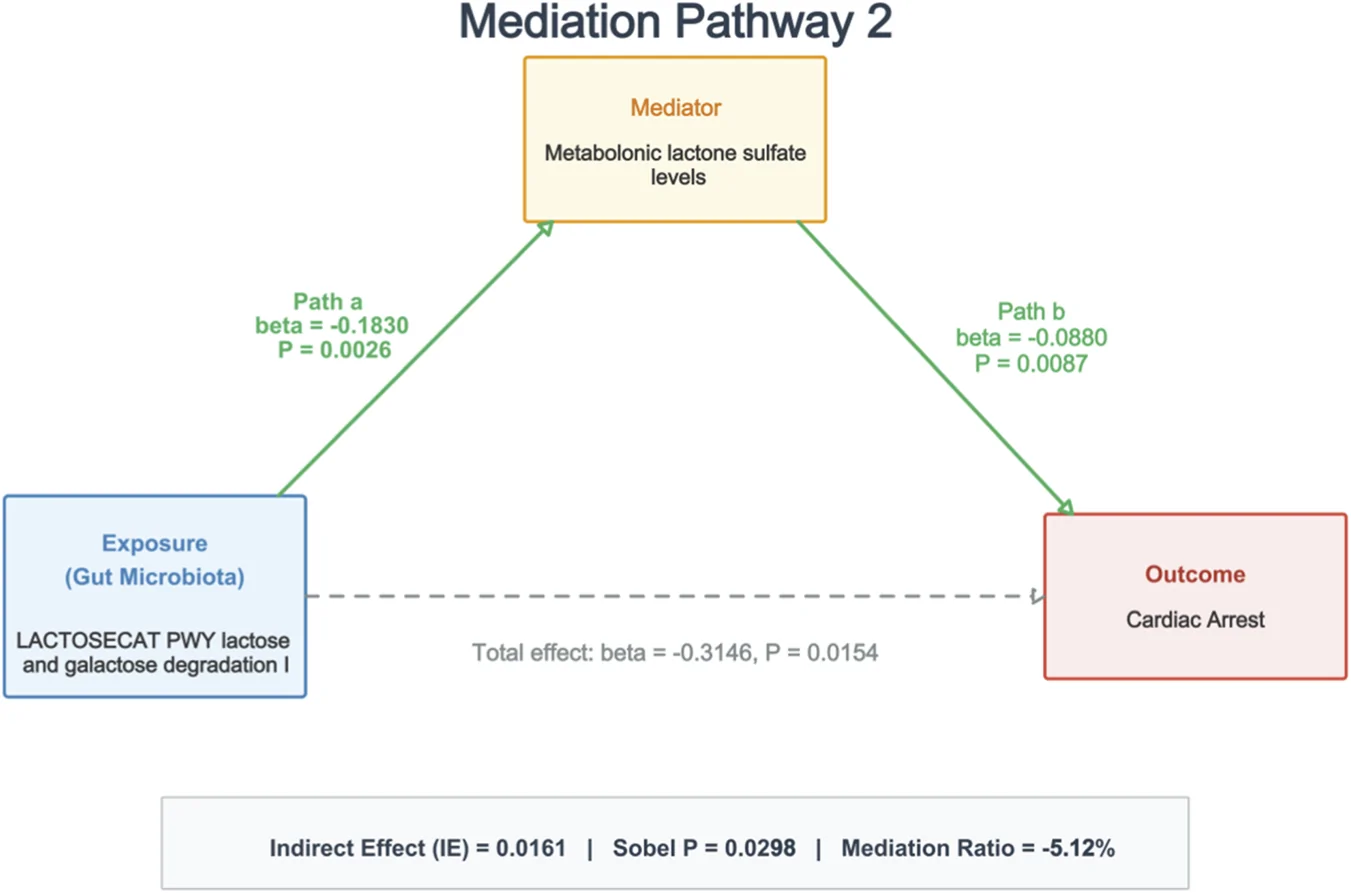
Representative exploratory mediation pathway: the LACTOSECAT microbial metabolic pathway and cardiac arrest through metabolonic lactone sulfate. The figure presents the exposure–mediator association, mediator–outcome association, total effect, and indirect effect. This pathway showed a nominally supported positive indirect effect (IE = 0.0161, Sobel P = 0.0298) within the exploratory mediation framework.

The distribution of indirect effects across exposure groups showed estimates clustered around zero with both positive and negative values (Supplementary Figures S3, S4). This pattern indicates heterogeneity among candidate mediation signals rather than proof of simultaneous risk-promoting and protective mechanisms.

We also observed differences in the number and mediator-category composition of candidate mediation pathways across exposures (Supplementary Figure S6). The LACTOSECAT pathway, *Escherichia coli*, and H. parainfluenzae were linked to relatively large numbers of candidate pathways; however, pathway counts alone should not be interpreted as evidence of biological importance or causal centrality.

Forest plots summarizing the candidate mediation pathways showed that most indirect effects were modest in magnitude. Only a subset exhibited comparatively stronger statistical support within the applied exploratory framework (Figure 9).

**FIGURE 9:**
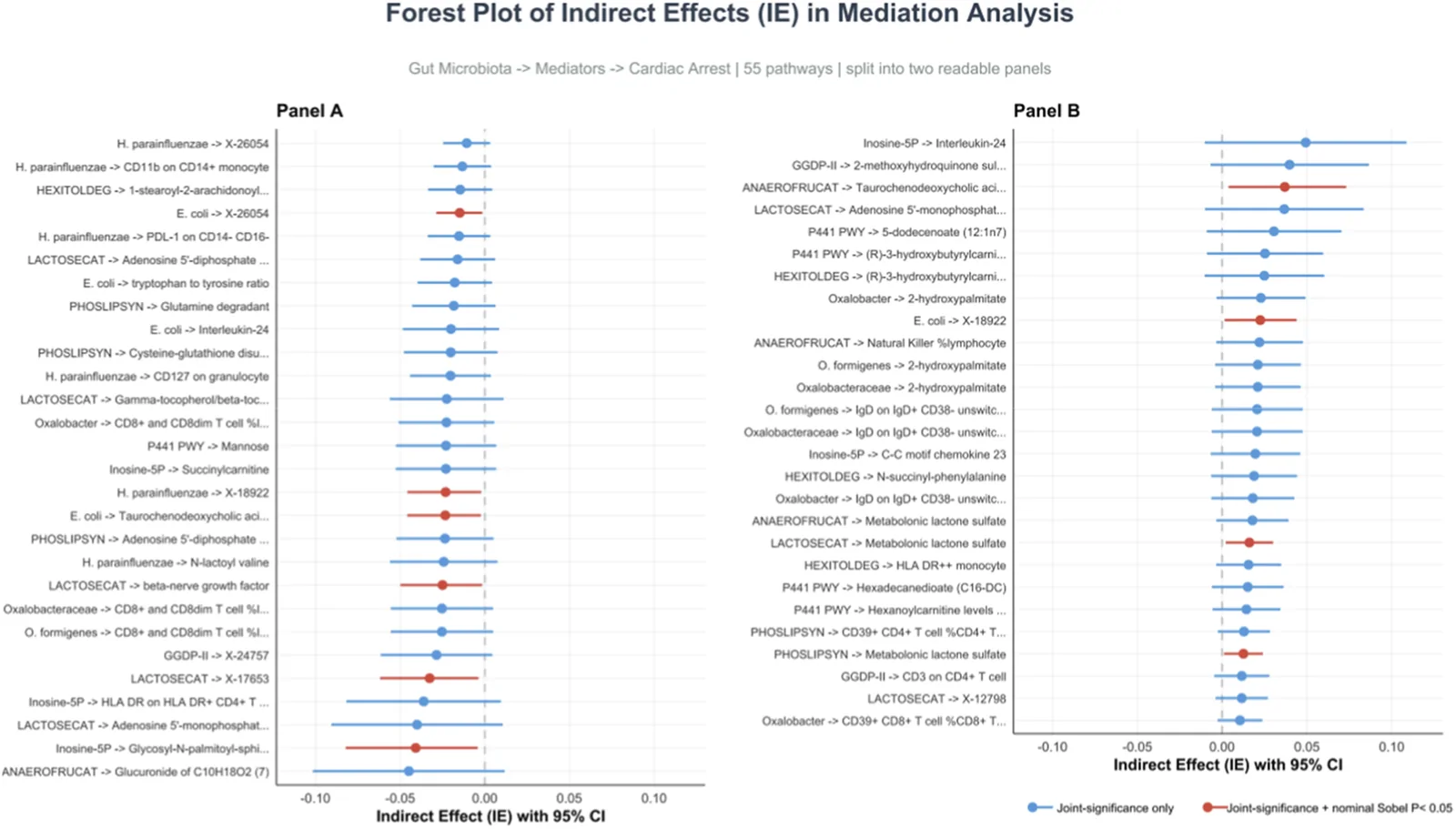
Forest plot of indirect effects (IEs) identified in the exploratory mediation analyses. The figure presents indirect effects and corresponding 95% confidence intervals for candidate mediation pathways. Red indicates pathways meeting the nominal Sobel test threshold (P < 0.05), whereas blue indicates pathways with Sobel P ≥ 0.05. The color coding represents nominal statistical support before correction for multiple testing and should not be interpreted as evidence of definitive mediation or causal mechanisms.

The 10 pathways additionally meeting the nominal Sobel P < 0.05 criterion are identified in Supplementary Table S15 and illustrated in Figures 7, 8 and Supplementary Figure S5.

Overall, the mediation analyses prioritized a network in which metabolic pathways represented the predominant candidate component, while immune and inflammatory signals provided additional candidate layers. These findings should be interpreted as hypothesis-generating associations requiring independent validation.

Detailed mediation pathway statistics are provided in Supplementary Table S15.

### 3.8 Exploratory fecal 16S rRNA sequencing analysis

To provide exploratory biological context for the Mendelian randomization findings, fecal 16S rRNA sequencing analyses were conducted in an independent exploratory clinical cohort comprising patients with cardiac arrest (n = 5) and healthy controls (n = 6). Representative descriptive outputs are shown in Figure 10, including Shannon diversity, Bray-Curtis principal coordinates analysis, genus-level compositional profiles, and regrouped predicted functional summaries.

**FIGURE 10:**
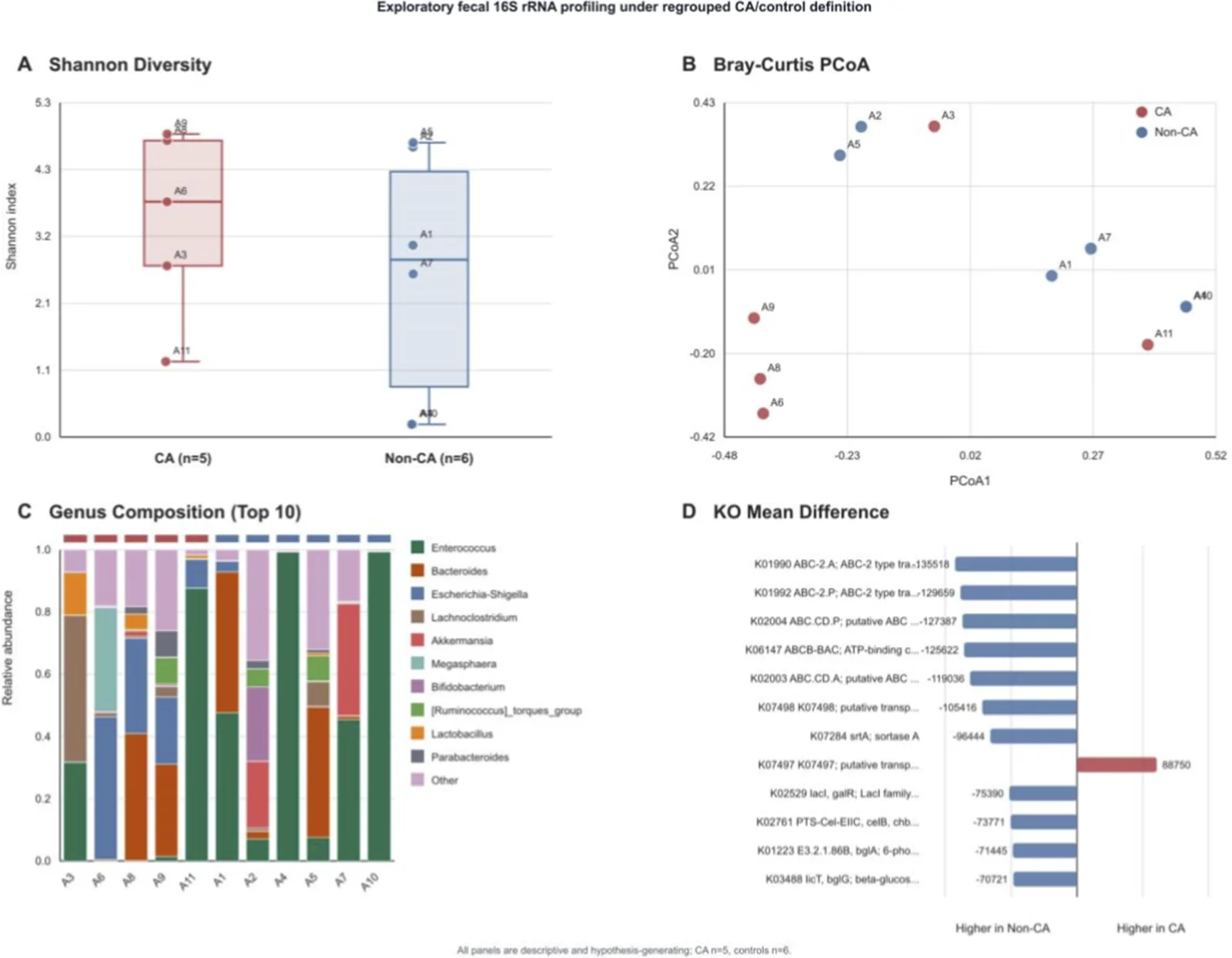
Exploratory fecal 16S rRNA profiling in patients with cardiac arrest and healthy controls. **(A)** Shannon diversity. **(B)** Bray-Curtis principal coordinates analysis (PCoA). **(C)** Sample-level genus composition in the regrouped cohort. **(D)** Exploratory differences in PICRUSt2-predicted KEGG Orthology profiles, shown as descriptive mean-abundance contrasts between the CA and control groups. Given the limited sample size (CA n = 5; controls n = 6), all panels should be interpreted as descriptive and hypothesis-generating.

Across the exploratory 16S cohort, Shannon diversity values and Bray-Curtis principal coordinates showed marked inter-individual heterogeneity and only partial overlap between groups, without supporting a strong inferential conclusion in this small sample set. At the compositional level, *Enterococcus*, *Bacteroides*, Escherichia-Shigella, Lachnoclostridium, and Akkermansia were among the dominant genus-level signals, while several taxonomic patterns highlighted by the genetic analyses appeared relatively enriched in part of the cardiac arrest group. These observations are descriptive and cannot establish directionality or validate the genetic associations.

Overall, these post-arrest observational microbiome patterns showed partial taxonomic overlap with selected features implicated by the genetic analyses; however, differences in taxonomic resolution, sampling time, and study design preclude direct validation or formal direction-of-effect comparison. Confirmation in larger prospective cohorts with prespecified microbiome analyses remains necessary.

Supplementary Table S16 Taxonomy-level absolute abundance profiles from the exploratory 16S rRNA sequencing cohort. The table summarizes curated abundance data across hierarchical taxonomic levels, from kingdom to species/combined lineage annotations, and provides the primary descriptive source data for the taxonomic composition and taxon-specific comparisons discussed in the main text.

## 4 Discussion

In this study, integrated genetic prioritization and exploratory microbiome profiling identified a candidate gut microbiota-associated immunometabolic framework related to CA susceptibility. Bidirectional two-sample Mendelian randomization analyses prioritized 14 gut microbial taxa or microbial metabolic pathways associated with CA risk. Among these 14 microbial traits, 12 formed at least one candidate mediation pathway meeting the nominal joint-significance criterion. PWY-1269 did not yield a candidate mediator meeting the pathway-a criterion, whereas the circulating proteins initially associated with genus *Haemophilus* did not meet the pathway-b criterion. The remaining 12 microbial exposures yielded 55 candidate mediation pathways involving 41 unique mediator traits. Metabolite-related pathways represented the majority of the prioritized signals, with additional candidate immune-cell and inflammatory pathways. The primary contribution of this study is the prioritization of biological layers that may connect gut microbial variation with CA susceptibility; it does not establish a single definitive mediator or a ranked causal framework.

The predominance of metabolite-related mediation suggests that metabolic signaling may represent a particularly informative intermediate layer for future investigation. Recurrent candidate mediators included bile acid-related metabolites, fatty acid and carnitine metabolism-related metabolites, sphingolipid-related metabolites, and redox-associated metabolites. These metabolic pathways are increasingly recognized as important regulators of host cardiovascular physiology through effects on energy metabolism, inflammatory signaling, and metabolic homeostasis (Ahmadmehrabi and Tang, 2017; Fiorucci and Distrutti, 2015). However, the present analyses do not establish tissue specificity, and the identified metabolites should not be interpreted as direct evidence of acting within cardiomyocytes or other specific cardiovascular cell types. Instead, these findings prioritize metabolic pathways that warrant further experimental evaluation.

Beyond metabolic pathways, immune cell phenotypes and inflammatory proteins provided an additional biological layer connecting gut microbial variation with CA susceptibility. Gut microbiota–host interactions have been increasingly recognized as important regulators of systemic immune homeostasis and inflammatory responses, which may contribute to cardiovascular disease development and progression (Belkaid and Hand, 2014) Recurrent signals involving CD8^+^ T-cell traits, monocyte-related phenotypes, dendritic cells, NK cells, IL-6, CCL23, and beta-NGF suggest that microbiota-associated alterations may influence CA susceptibility partly through immune regulation and inflammatory signaling. Rather than supporting a single linear pathway, these findings are more consistent with a model of coordinated immunometabolic crosstalk, in which metabolic and immune states may interact under conditions of cardiovascular stress. The specific tissue-level mechanisms involving myocardial, vascular, or circulating immune-cell responses remain to be determined through dedicated experimental studies.

The exploratory fecal 16S rRNA sequencing cohort provided limited, descriptive post-arrest biological context. Although global diversity indices and ordination analyses did not demonstrate robust separation between CA patients and controls, descriptive enrichment patterns involving Gammaproteobacteria-, Enterobacteriaceae-, and Escherichia-Shigella-related signals were observed in a subset of CA patients. These findings should not be considered directional concordance or validation of the genetic prioritization because of differences in taxonomic resolution, sampling time, and study design. Larger prospective cohorts will be required to determine reproducibility and clinical relevance.

From a cell and developmental biology perspective, the most actionable outcome of this study is the prioritization of experimentally testable biological pathway classes rather than the establishment of a completed mechanistic model. The metabolite-dominant signals identified in the mediation analyses provide a rationale for future studies examining whether specific microbial-derived metabolic alterations influence cellular energetics, mitochondrial stress responses, redox balance, membrane lipid composition, or inflammatory activation. Microbial metabolites have been increasingly recognized as important regulators of host cellular metabolism, immune responses, and mitochondrial function, providing mechanistic links between microbial variation and host physiology (Koh et al., 2016; Rooks and Garrett, 2016). Such questions can be investigated through targeted metabolomics in larger human cohorts, metabolite perturbation experiments in cardiomyocytes or immune cells, and animal models that allow direct assessment of tissue-specific responses relevant to CA susceptibility and progression.

From a translational perspective, the identified gut microbiota-associated pathways may provide candidate biomarkers and potential intervention targets for future CA risk assessment. Circulating metabolites, immune phenotypes, and inflammatory factors repeatedly emerging within the mediation framework may contribute to multidimensional risk models when integrated with established cardiovascular risk factors. Previous studies have highlighted the potential of gut microbiota-derived metabolites and inflammatory signatures as biomarkers reflecting cardiometabolic risk and disease progression (Loscalzo, 2013; Tang et al., 2013). Furthermore, microbiota-targeted strategies, including dietary modulation, microbial metabolite regulation, or other approaches aimed at restoring host–microbiota homeostasis, represent potential avenues for future investigation. Dietary interventions and microbiota-directed approaches have been shown to influence microbial composition and host metabolic pathways, although their clinical efficacy for cardiovascular prevention remains under active investigation (Lim, 2013; Zhang et al., 2015). However, whether modifying gut microbial composition or related metabolic pathways can reduce CA susceptibility remains unknown and requires validation in prospective clinical studies and experimental models.

Several limitations should be considered when interpreting these findings. First, although MR can reduce confounding and reverse causation under its core assumptions, it remains an instrument-based genetic approach and cannot replace direct molecular or cellular validation. Second, the 55 candidate mediation pathways were retained using nominal significance criteria for both pathway a and pathway b from a larger, high-dimensional upstream screening space. Formal multiple-testing correction across the complete set of initially evaluated exposure–mediator combinations was not performed. Therefore, false-positive findings remain possible, and all pathway-level results should be considered exploratory and require independent replication. Substantial sample overlap was considered unlikely based on the reported cohort sources; however, participant-level overlap among all exposure, mediator, and outcome GWAS datasets could not be formally quantified or completely excluded. Selection of microbial traits based on the initial MR associations may have introduced winner’s curse and selection-related bias. Collider bias may also arise when analyses are conditioned on genetically selected exposure or mediator signals. These issues should be considered when interpreting the exploratory mediation estimates. Third, a relaxed instrumental variable threshold was required for some gut microbial traits because of limited available genetic instruments, leaving potential weak-instrument bias despite sensitivity analyses. Fourth, the CA phenotype was derived from biobank-based clinical datasets and reflects genetic liability to CA risk rather than a narrowly defined arrhythmic subtype or cell-specific endpoint. Formal Steiger directionality testing was not performed; therefore, the asymmetry between the forward and reverse MR findings cannot establish causal direction or confirm the assumed mediation sequence. Fifth, the post-ROSC 16S cohort was small and descriptive; acute illness, treatment, medication exposure, nutrition, and sampling time may have influenced the observed microbiome. Sixth, comprehensive sensitivity analyses were not performed for every exposure–mediator–outcome combination, so the robustness of individual mediation estimates remains uncertain. In addition, the product-method calculation assumed zero covariance between βa and βb. If this assumption does not hold, the precision of the estimated indirect-effect standard errors and confidence intervals may be affected. Finally, most source GWAS datasets were derived primarily from individuals of European ancestry, which may limit generalizability to other populations. These limitations highlight the need for larger prospective, multi-ethnic cohorts and dedicated mechanistic studies.

In summary, this study provides genetic and exploratory microbiome evidence prioritizing potential gut microbiota-associated immunometabolic pathways related to CA susceptibility. Rather than identifying a single causal mediator, the analyses highlight candidate metabolic, immune, and inflammatory layers for future investigation. The post-ROSC 16S findings provide descriptive context but not independent validation, and the mediation results should be interpreted as exploratory candidate signals. The proposed pathways should be tested in larger prospective cohorts and targeted cellular or experimental systems.

## 5 Conclusion

Using a bidirectional two-sample Mendelian randomization and mediation-analysis framework, this study prioritizes candidate associations linking gut microbial traits with cardiac arrest risk. Metabolite-related signals represented the predominant candidate intermediary layer, with additional immune and inflammatory candidates. The exploratory post-ROSC 16S analysis provided descriptive microbiome context but did not establish pre-arrest risk or causal direction. These findings generate hypotheses for future mechanistic and translational research.

## Data Availability

The original contributions presented in the study are included in the article/Supplementary Material, further inquiries can be directed to the corresponding authors.
