# Supplementary material for: Gut microbial immunometabolic pathways associated with cardiac arrest risk: genetic evidence and exploratory fecal microbiome profiling
Source: Front Cell Dev Biol. 2026 Oct 13;14:1931987. doi: 10.3389/fcell.2026.1931987 (PMC13710367; doi:10.3389/fcell.2026.1931987)
Supplement: Supplementary file 1 [file DataSheet1.docx]

**Supplementary Figure S1. Core assumptions of Mendelian randomization.** Schematic representation of the three instrumental-variable assumptions: relevance, independence, and exclusion restriction. Genetic variant(s) must be associated with the exposure, must be independent of confounders of the exposure–outcome association, and must affect the outcome only through the exposure. Solid arrows indicate permitted associations; dashed paths crossed in red indicate associations that should be absent.

**Supplementary Figure S2. Reverse Mendelian randomization estimates for genetic liability to cardiac arrest and gut microbial traits.** Forest plot summarizing reverse Mendelian randomization estimates with genetic liability to cardiac arrest as the exposure and the evaluated gut microbial traits as outcomes. Red squares indicate odds-ratio estimates and horizontal lines indicate 95% confidence intervals; the dashed vertical line denotes the null value (OR = 1). The numbers of single-nucleotide polymorphisms (nSNP), association P values, MR-Egger pleiotropy P values, and Cochran’s Q heterogeneity P values are shown. Rows correspond to the gut microbial traits reported in Supplementary Table S14.

**Supplementary Figure S3. Distribution of indirect effects according to microbial exposure type.** Violin and box plots show the distribution of indirect-effect estimates for microbial metabolic pathways and taxonomic exposures (species, genus, or family). Individual points represent candidate mediation pathways. Blue points denote pathways meeting the nominal joint-significance criterion only, whereas red points denote pathways additionally meeting the nominal Sobel P < 0.05 criterion. These classifications are based on unadjusted nominal thresholds and do not represent evidence surviving correction for multiple testing.

**Supplementary Figure S4. Distribution of indirect effects across the 12 microbial exposures included in the mediation analysis.** Violin and box plots summarize indirect-effect estimates for each microbial exposure; each point represents one candidate mediation pathway. Circles denote pathways meeting the nominal joint-significance criterion only, and triangles denote pathways additionally meeting the nominal Sobel P < 0.05 criterion. The horizontal dashed line indicates an indirect effect of zero. All pathway-level findings are exploratory and were not interpreted as definitive mediation after multiple-testing correction.

**Supplementary Figure S5. Additional candidate mediation pathways meeting the nominal Sobel criterion.** Path diagrams for mediation pathways 3–10, complementing the two representative pathways shown in main-text Figures 7 and 8. For each pathway, path a represents the genetically predicted microbial exposure–mediator association, path b represents the separately estimated mediator–cardiac arrest association, and the dashed horizontal arrow represents the total exposure–outcome association. The indirect effect was calculated as βa × βb. Sobel P values and mediation ratios are displayed below each diagram. Negative mediation ratios indicate inconsistent mediation or suppression patterns and should not be interpreted as conventional percentages mediated. All displayed Sobel P values are nominal and unadjusted for multiple testing.

**Supplementary Figure S6. Distribution of candidate mediation pathways by microbial exposure and mediator category.** Grouped bars show the numbers of candidate mediation pathways involving circulating metabolites, inflammatory proteins, and immune-cell phenotypes for each microbial exposure. All 55 pathways met the nominal joint-significance criterion, defined as P < 0.05 for both pathway a and pathway b. No formal multiple-testing correction was applied; therefore, these pathways should be interpreted as exploratory and hypothesis-generating.
